# The Role of Acidosis in the Pathogenesis of Severe Forms of COVID-19

**DOI:** 10.3390/biology10090852

**Published:** 2021-08-31

**Authors:** Yury D. Nechipurenko, Denis A. Semyonov, Igor A. Lavrinenko, Denis A. Lagutkin, Evgenii A. Generalov, Anna Y. Zaitceva, Olga V. Matveeva, Yegor E. Yegorov

**Affiliations:** 1 Laboratory DNA-Protein Recognition, Engelhardt Institute of Molecular Biology, Russian Academy of Sciences, Moscow 119991, Russia; 2 Institute of Molecular Medicine and Pathobiochemistry, Voyno-Yasenetsky Krasnoyarsk State Medical University, Krasnoyarsk 660022, Russia; semenov@ibp.ru; 3 Institute of Biophysics Siberian Branch of Russian Academy of Sciences, Krasnoyarsk 660036, Russia; 4 Department of Human and Animal Physiology, Faculty of Medicine and Biology, Voronezh State University, Voronezh 394018, Russia; lavrinenko_ia@bio.vsu.ru; 5 Department of Biological and Medical Physics, Moscow Institute of Physics and Technology, Dolgoprudny 141701, Russia; lagutkin.da@phystech.edu; 6 Department of Biophysics, Faculty of Physics, Lomonosov Moscow State University, Moscow 119991, Russia; generals1179@gmail.com; 7 Laboratory of Medical Analytical Methods and Devices, Institute for Analytical Instrumentation of the Russian Academy of Sciences, St. Petersburg 198095, Russia; anna@da-24.ru; 8 Sendai Viralytics LLC, Acton, MA 117261, USA; olga.matveeva@gmail.com; 9 Laboratory of Cellular Bases for the Development of Malignant Diseases, Engelhardt Institute of Molecular Biology, Russian Academy of Sciences, Moscow 119991, Russia

**Keywords:** SARS-CoV-2, COVID-19, acidosis, hypoxia, saturation, Bohr effect, lactate, pH

## Abstract

**Simple Summary:**

Recently, several studies have shown that acidosis, which is increased acidity in the blood and other body tissues, is often associated with severe COVID-19. In this article, we look at the mechanisms and consequences of acidosis that can lead to an exacerbation of COVID-19. We want to draw the attention of readers to the threshold values of such disease characteristics as hypoxia and acidosis, which are associated with a sharp deterioration in the patient’s condition. Hypoxia and acidosis mutually reinforce each other according to the principle of a vicious cycle (that is, they are involved in a system of positive feedbacks). Elevated blood lactate (lactic acid) levels are associated with poor clinical outcomes in COVID patients. As a practical recommendation, we propose to pay more attention to the prevention of acidosis, including in the early stages of the disease, when the adjustment of homeostasis requires less effort and is less risky.

**Abstract:**

COVID-19 has specific characteristics that distinguish this disease from many other infections. We suggest that the pathogenesis of severe forms of COVID-19 can be associated with acidosis. This review article discusses several mechanisms potentially linking the damaging effects of COVID-19 with acidosis and shows the existence of a vicious cycle between the development of hypoxia and acidosis in COVID-19 patients. At the early stages of the disease, inflammation, difficulty in gas exchange in the lungs and thrombosis collectively contribute to the onset of acidosis. In accordance with the Verigo-Bohr effect, a decrease in blood pH leads to a decrease in oxygen saturation, which contributes to the exacerbation of acidosis and results in a deterioration of the patient’s condition. A decrease in pH can also cause conformational changes in the S-protein of the virus and thus lead to a decrease in the affinity and avidity of protective antibodies. Hypoxia and acidosis lead to dysregulation of the immune system and multidirectional pro- and anti-inflammatory reactions, resulting in the development of a “cytokine storm”. In this review, we highlight the potential importance of supporting normal blood pH as an approach to COVID-19 therapy.

## 1. Introduction

One of the most important conditions for the adaptation and survival of an organism is the maintenance of the acid-base balance of the internal environment. In metabolic processes, an excess of acid is constantly formed, which can be removed from the body with the help of the lungs (removal of carbon dioxide with respiration) and the kidneys (the release of protons into the urine). Changes in blood pH are smoothed out by several buffer systems: hemoglobin, bicarbonate, phosphate, and plasma proteins. Acidosis, a decrease in blood pH, can occur for a variety of reasons. A distinction is made between respiratory acidosis (the main cause of which is associated with difficulty in removing carbon dioxide by the lungs) and metabolic acidosis, which can have various causes such as loss of bicarbonate, elevated acid production, and reduced ability of the kidneys to excrete excess acids. There are two variants of metabolic acidosis, which are lactic acidosis and ketoacidosis.

Recently, several studies have provided evidence that acidosis is often associated with a severe form of COVID-19 [1,2,3]. In this paper, we consider the mechanisms and consequences of the acidosis, which can lead to aggravation of the course of COVID-19 disease. We also draw attention to threshold changes that worsen the condition of patients. Particular attention is paid to lactic acidosis and to the lactate molecule, since, in addition to performing metabolic functions, lactate significantly affects the functioning of the immune system. We believe that studying this impact is important for understanding COVID-19 pathophysiology.

It has been shown that the blood lactate levels were significantly higher in COVID-19 hospitalized patients than ambulatory patients, and among hospitalized patients, lactate levels were the highest in non-survivors [4,5,6]. Thus, blood lactate is an independent predictor correlated with disease severity and associated with high in-hospital mortality. As a practical recommendation, we propose to pay more attention to the prevention of acidosis, including the early stages of the disease when adjusting homeostasis requires less effort and carries less risks.

## 2. Risk Group and Acidosis

The risk group for developing severe pneumonia in COVID-19 includes people over 65, patients with diabetes, cardiovascular diseases, obesity, cancer, chronic obstructive pulmonary disease (COPD), chronic kidney disease and pregnancy [7,8,9,10]. People with these conditions are more vulnerable to metabolic acidosis during COVID-19 see, for example, J. Li et al. [11]. We think that acidosis exacerbates the severity of COVID-19, being the common denominator in these groups of people.

It manifests itself as lactic acidosis, ketoacidosis, and is less commonly associated with base loss [12,13,14]. The literature frequently reports an increased risk of ketoacidosis in COVID-19 patients with diabetes mellitus, and diabetes is a risk factor for developing severe and critical forms of COVID-19 [15,16,17]. Moreover, COVID-19 infection causes ketosis or ketoacidosis, in addition to induction of diabetic ketoacidosis [11]. It was hypothesized that SARS-CoV-2 infection may either precipitate a new type of diabetes mellitus by a direct damage of pancreatic cells or indirectly induce some other pathophysiological diabetes-related mechanisms [18,19]. However, the focus of this article is on lactic acidosis, the pathophysiological effects of which are less well understood. It is possible that respiratory acidosis associated with high blood CO_2_ levels and metabolic acidosis in the form of ketoacidosis and lactic acidosis may work synergistically when the body’s natural buffer systems are exhausted.

Metabolic acidosis in COVID-19 usually does not develop immediately, but only after a significant time due to the gradual depletion of the body’s resources.

Normally, the level of lactate in the blood does not exceed 2 mM. However, in overweight people, as well as under certain conditions in pregnant women, an increase in the concentration of lactate and ketone bodies in the blood is observed [20]. High concentrations of lactate, as well as ketone bodies in the blood of overweight people and, certain pregnant women are detected, including before the onset of respiratory diseases, and the occurrence of pneumonia only aggravates the already existing acidosis.

Next, we will try to prove that metabolic acidosis is the cause and effect of many phenomena characteristic of the severe course of COVID-19.

## 3. Factors Determining the Disruption of Gas Exchange in COVID-19

The pneumonia that develops with COVID-19 leads to disruption of gas exchange in the lungs, which provokes the development of hypoxia. Hypoxia induces the transition to anaerobic metabolism and increases lactate production. The hypoxic disorder is observed in several diseases (mainly with respiratory involvement). However, this disorder is one of the main defining features of COVID-19. Severe Acute Respiratory Syndrome is abbreviated as SARS and is included in the name of the SARS-CoV-2 virus that causes COVID-19.

**Figure 1:**
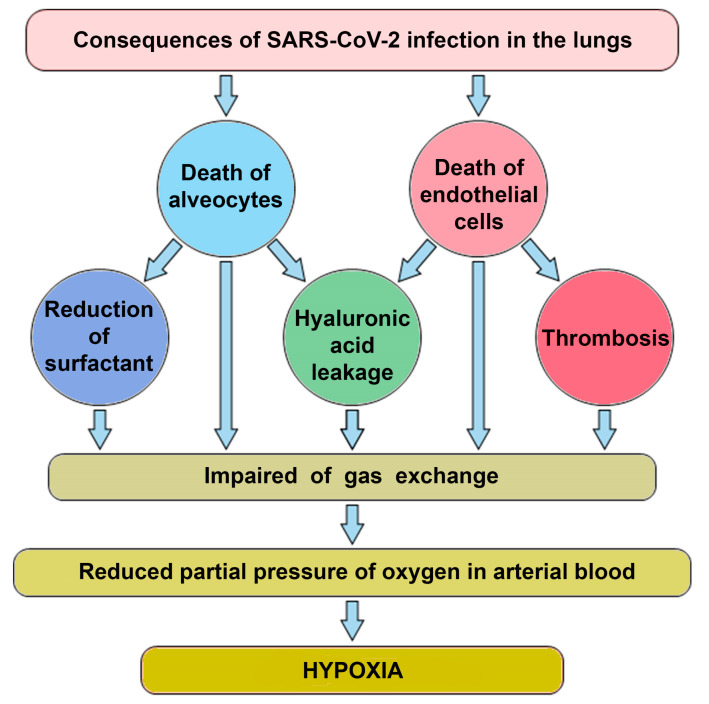
A diagram illustrating the relationship between viral lung damage and the occurrence of hypoxia.

There are at least four factors contributing to the disruption of gas exchange in COVID-19 (Figure 1).

1. The lytic phase of the virus leads to the death of type 2 alveolocytes, which disrupts the structure of the alveoli [21]. The collapse of alveoli is facilitated by a decrease in the production of surfactant in alveolocytes, which leads to a change in surface tension. As a result, the changes in air pressure in the lungs do not lead to compression and expansion of the alveolar vesicles, the dead volume of lungs increases and gas exchange decreases;
2. COVID-19 is frequently manifested by thrombosis in pulmonary capillaries, which prevents the transfer of oxygen [22];
3. Intensive production of the extracellular matrix (ECM), rich in hyaluronic acid, is designed to “seal” areas of extensive lung damage to prevent general lung collapse. As a side effect of this process, a part of functional alveoli is filled with ECM and switched off from gas exchange [23];
4. Another of the mechanisms limiting gas exchange in patients with COVID-19 is a change in the properties of the erythrocytes themselves. The virus can infect erythroid precursor cells. This, in turn, leads to a greater production of immature red blood cells, followed by their massive release into the bloodstream and a corresponding drop in hemoglobin levels [24].

## 4. Inflammation and Acidosis

Any intensive inflammation contributes to the increase of acidosis, especially local inflammation. This is due to three factors. First, gas exchange takes place mainly in small vessels. The increased metabolic activity that results from inflammation requires oxygen, which is limited. Therefore, when small vessels are damaged, hypoxia and increased metabolic activity of infiltrating leukocytes shifts metabolism toward glycolysis, which leads to the accumulation of lactate [25]. Second, in the process of oxidative burst that often accompanies inflammation, there is a massive production of protons by neutrophils [26]. Third, in case of concomitant bacterial infection, short-chain fatty acids accumulate [27,28].

**Figure 2:**
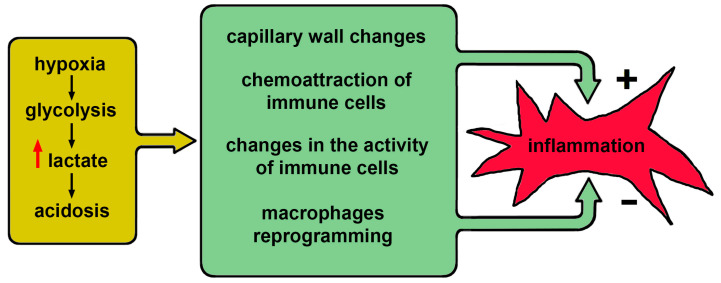
A diagram illustrating the complex relationship between hypoxia and acidosis on the one hand and the development of inflammation.

The above effects are not specific to COVID-19 but considering them in a single system allows us to represent the overall development of this disease as a set of direct and inverse relationships between inflammation and acidosis (see Figure 2).

## 5. Low pH and Hypoxic Conditions Disrupt the Regulation of the Innate and Adaptive Immune Response

Prolonged hypoxia leads to acidosis, which promotes chronic inflammation both locally and in a generalized manner due to the production of large amounts of pro-inflammatory cytokines and the recruitment of immune cells. All major immune cell functional programs and communication pathways are affected.

Hypoxia at the site of inflammation causes macrophage infiltration and switching of oxidative phosphorylation to glycolysis. It happens mainly due to increased metabolic activity and enhanced macrophage expression of hypoxia-inducible factor 1α (HIF1α) [29]. This factor is a transcriptional regulator and is an essential mediator of oxygen homeostasis. It can mediate both physiological and pathophysiological responses to hypoxia [29]. It is believed that HIF1α and metabolites of the glycolytic pathway contribute to the acidification of the microenvironment through upregulation of the production and release of lactate by macrophages [30,31]. Lactate itself can reduce pro-inflammatory action of macrophages via direct binding to histones and delayed enhancing of the expression of homeostatic genes [32]. It was proposed that histone lactylation after cell activation serves as a “lactate clock” to promote late-phase reprogramming to a homeostatic phenotype [30].

However, lactate increases endothelial permeability for neutrophils and attracts them to the site of inflammation by elevating the levels of chemokines. Lactate can also enhance the release of growth factor, which stimulates the bone marrow to produce new granulocytes. Lactate serves as an inhibitor of macrophage proinflammatory function, nevertheless, it is a potent chemoattractant for granulocytes, which causes the release of proinflammatory effector molecules by neutrophils [33]. Therefore, proinflammatory signals may prevail causing hyperinflammation.

Hypoxia is likely to be an important factor contributing to the development of hyperinflammation seen in severe COVID-19. Acidosis influences the functioning of immune cells and further stimulates the development of inflammation. Short-term hypoxia and local acidosis are prominent features of the inflammatory microenvironment that regulate key transcription factors in both innate and adaptive immunity but prolonged exposure of these features on immune cells leads to dysregulation of responses to antigens. This dysregulation causes the transformation of physiological reactions into pathophysiological ones.

Thus, prolonged hypoxia leads to acidosis, and a combination of these factors causes hyperactivation of dendritic cells and dysregulation of cytokine response. These affect antigen internalization and presentation. Hypoxia and lactic acidosis attract macrophages, leading to the early pro-inflammatory response with delayed homeostatic reprogramming and potently stimulate neutrophil migration and activation. All these factors are likely to synergistically contribute to the development and maintenance of the state of hyperinflammation, which is characteristic of severe forms of COVID-19.

## 6. Bohr Effect—Basic Threshold Effect Linking Acidosis and Hypoxia

**Figure 3:**
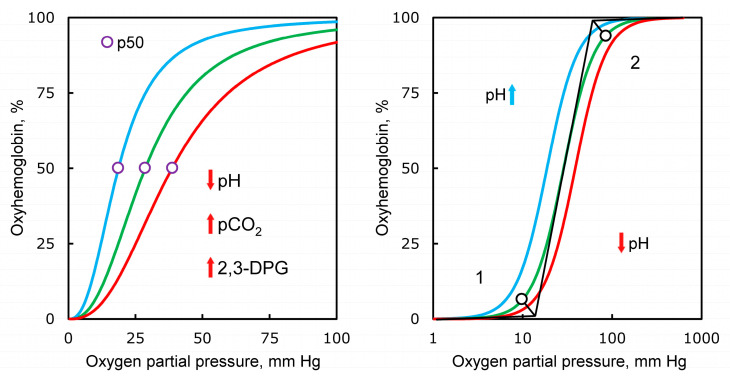
Different factors influencing oxygen saturation of hemoglobin. During pH change from 7.4 to 7.2, saturation decreases two times. The concentration of 2,3-diphosphoglycerate provides saturation of hemoglobin with oxygen, along with CO_2_ pressure. pH, negative of the base 10 logarithm of the activity of the H^+^ ion; pCO_2_, partial pressure of carbon dioxide gas; 2,3-DPG, 2,3-diphosphoglycerate; p50, oxygen tension at 50% hemoglobin saturation.

Severe pneumonia in COVID-19 is accompanied by a drop in blood oxygen saturation. At the same time, the first stage of saturation reduction proceeds relatively smoothly, it can develop for several days. The subsequent drop in saturation and the occurrence of oxygen deficiency are already occurring rapidly and require hospitalization, oxygen support and intensive care. We believe that the threshold effect of a rapid decrease in saturation is directly related to acidosis and is explained by the Bohr effect, observed in the analysis of oxygen binding by hemoglobin [34]. The curve of hemoglobin oxygen saturation in relation to oxygen partial pressure has an S shape (Figure 3). As the partial pressure of oxygen in the blood increases, its binding to hemoglobin initially practically does not change until it reaches a certain boundary value (first inflection point), then increases sharply—and then reaches a plateau reflecting saturation (second inflection point).

The local oxygen concentration required to maintain normal cell metabolism in peripheral tissues is determined by the pH in these tissues. With intensive metabolism, leading to a decrease in pH, hemoglobin gives up O_2_ more easily, while binding excess protons, thereby providing efficient transport of oxygen from the lungs to tissues, and the transport of carbon dioxide (mainly in the form of bicarbonate) in the opposite direction. This system of metabolites regulation with negative feedback is based on a cooperative pH-dependent change in the conformation of hemoglobin and for over 100 years has been known as the Verigo-Bohr effect (hereinafter referred to as the “Bohr effect”) [35,36,37,38].

A slight deviation of blood acidity from the physiological norm can significantly change the ability of hemoglobin to bind oxygen. It should be emphasized that the Bohr effect is a key element in the regulation of gas exchange in humans and many animals. A decrease in blood plasma pH from 7.4 to 7.2 leads to a twofold reduction in the amount of O_2_ that Hb can bind at a partial pressure of oxygen in the tissue fluid of the order of 20–40 mm Hg. Therefore, during oxygenation, it is critically important for the body to maintain the optimal pH value in the blood plasma of alveolar capillaries.

An important regulator of oxygen transport is 2,3-diphosphoglycerate (2,3-DPG). The formation of lactate is accompanied by the accumulation of 2,3-DPG (Rappoport shunt) with the migration of the latter into erythrocytes. 2,3-DPG is incorporated into hemoglobin by forming a salt bridge between the β-subunits of the heterotetramer, which prevents oxygen re-binding. This is another mechanism that allows you to regulate the transport of oxygen, in essence, it complements the regulation with the help of the Bohr effect [39,40].

## 7. Physiological Aspects of the Compensatory Mechanisms of Acidosis

A significant contribution to the acid-base balance of the body made by the activity of some internal organs, such as, for example, the respiratory center of the brain, which is essentially a physiological system for monitoring pH and controlling respiration [34]. When chemical stimuli, such as hypoxia and hypercapnia (elevated CO_2_ level in the blood), are recognized by chemoreceptors, the respiratory center increases the flow of impulses to the respiratory motor neurons, which results in increased ventilation. Arterial hypocapnia (low CO_2_ levels), on the contrary, causes a decrease in ventilation [41,42]. In the case of SARS-CoV-2 virus infection, impaired CO_2_ excretion through exhaled air leads to stimulation of hyperventilation to reduce CO_2_ concentration. If hypoventilation occurs, i.e., alveolar ventilation is insufficient to eliminate CO_2_ produced in the body, a hypercapnic shift occurs, and the partial pressure of carbon dioxide gas increases [41,43,44].

Metabolic acidosis causes rapid breathing and a decrease in the concentration of carbon dioxide in the lungs. Thus, metabolic acidosis can be compensated to an extent by respiratory alkalosis. If a decrease in pH and a drop in blood oxygen saturation has already occurred, then the body’s compensatory capabilities to regulate acidosis have been exhausted [45]. Apparently, this often happens in case of SARS-CoV-2 infection since a drop in pH and blood oxygen saturation are characteristic features of the severe course of COVID-19.

The severe course of the COVID-19 disease occasionally leads to damage to the carotid bodies, which are the “sensor of blood saturation” in the human body [46]. Such damage leads to the loss of the natural mechanism of restoration of oxygen saturation by increasing the respiratory rate. This sensors’ damage is another trigger, which is a consequence of the severe course of the disease, in which hypoxia becomes prolonged and dangerous, although it is subjectively perceived easily (the so-called state of “happy hypoxia”). The main danger of this condition is the development of encephalopathy. We do not know to what extent this condition generates acidosis, but the recorded oxygen saturation levels in such patients are well below 80%.

## 8. COVID-19-Associated Coagulopathy May Contribute to Hypoxia and Acidosis

Disseminated intravascular hypercoagulation is a severe complication of COVID-19, exacerbating hypoxia and acidosis. The pathophysiology of this general clotting disorder is different from that of septic-related complications [47].

SARS-CoV-2 affects not only alveolocytes, but indirectly damages endothelial cells. This leads to a disruption in the endothelium’s functioning, vasoconstriction, pro-inflammatory state, and induction of a procoagulant shift in the hemostatic balance [48].

Migration of monocytes to the affected endothelium induces the activation of coagulation cascades and platelet aggregation, resulting in disseminated intravascular coagulation (DIC) syndrome-like complications [49,50].

### Acidosis May Dysregulate the Balance between Coagulation and Fibrinolysis

Hypercoagulation provokes microthrombosis in the pulmonary vessels and then can lead to systemic thrombosis [50]. In the lungs, blood clotting disrupts gas exchange and promotes hypoxia throughout the body. In most body tissues, blood clotting causes acute hypoxia and significantly upregulates lactate production, therefore local lactate concentration increases. This lactate concentration can be further increased by the migration of activated neutrophils and monocytes through the lesions in the endothelium. However, it has been shown that acidosis (pH 6.8–7.4), including the one resulting from increased lactate concentration, causes a reversible decrease in blood clotting [51,52].

The localized decrease in pH and depletion of coagulation factors at the advanced stages of COVID-19 disease appear to lead to anticoagulant effects, thus contributing to the cyclic switch of procoagulant state to secondary hyperfibrinolysis and back compensatory hypercoagulability [50,53]. Decompensation at one of these stages leads to a critical state of the patient, reflected in the coagulation tests [47,53].

The body’s ability to compensate for acidosis is one of the critical factors, which determine its resistance to changing phases of coagulation and fibrinolysis. Excessive lactate can break the fragile balance. For example, it has been shown that an increased concentration of lactate is a predictor of an extremely unfavorable course of non-COVID DIC and pulmonary embolism [54,55,56].

Thus, thrombosis can lead to acidosis in two ways at once (see Figure 1): it leads directly to hypoxia (the first pathway, the extreme arrow at the bottom left) and it leads to a decrease in gas exchange and, through it, to acidosis. The formation of blood clots is also a threshold phenomenon that leads to a sharp deterioration in the patient’s condition.

## 9. Diarrhea Caused by Viral Inflammation Contributes to Acidosis

Sometimes the disease is accompanied by intestinal upset, and this is associated with a further severe course of the disease [57]. We believe that the relationship between diarrhea and severe disease is directly explained by acidosis, and we will try to demonstrate this.

It is known that the SARS-CoV-2 virus can infect the intestines, as cells with ACE2 are present in large numbers there [58]. In this regard, diarrhea results in an abrupt and significant loss of bicarbonate [59]. After depletion of the bicarbonate buffer, the body cannot contain the developing acidosis. The loss of bicarbonate because of diarrhea can abruptly bring the onset of acidosis closer and at the level of the whole organism.

## 10. The Special Role of Lactate Accumulation in COVID-19

Lactate is an important regulatory molecule; therefore, an increase in the concentration of lactate can affect many processes in the body during the disease process.

The attitude to lactate over the past 20 years has undergone significant changes: from a “waste” metabolic product, lactate has come to be regarded as the basis of energy metabolism. Lactic acidosis corresponds to lactate concentration above 2 mM. This condition is common in patients with type 2 diabetes, cardiovascular disease, and in patients with acute inflammation. Prolonged staying in this condition depletes carbohydrate and glycogen stores in the body.

For patients with COVID-19, an increase in lactate concentration above 2 mM is associated with an increased likelihood of fatality [6,60].

Having exhausted carbohydrate depots, the body can follow the path of synthesizing carbohydrates from amino acids, which will lead to the need to neutralize and remove nitrogen metabolism products (ammonia and urea) and thus increase the load on the kidneys. It is necessary to pay attention to the existence of two thresholds: at a concentration of lactate above 2 mM lipolysis is turned off, at 4 mM, the body is not able to maintain for a long time the equilibrium between the formation and removal of lactate [61,62].

In 2020, an article was published in which lactate was called the “ugly duckling” of energy metabolism [63]. Lactate exerts its effects at different levels: through a special cell surface receptor, or after entering (leaving) cells (both processes are associated with the transport of a proton, therefore they depend on pH). Lactate participates in redox regulation. In the conditions of glucose depletion, lactate plays a role of a reserve source of energy.

Lactate, being a product of glycolysis, signals the body about impending hypoxia by increasing its concentration. Recently, it has been suggested that lactate contributes to the reprogramming of macrophages into the M2 type—angiogenic, aimed at restoring damage, and not at fighting infection—thus, the body’s resources needed to fight infection are diverted to extraneous activities and, as a result, are depleted faster [64,65,66,67]. It is believed that lactate is generally an immunosuppressive agent that acts on all types of immune cells [68].

Despite the general anti-inflammatory nature of the action of lactate, an increase in its concentration can induce neutrophil NETosis, which leads to a noticeable increase in the amount of extracellular DNA and increases inflammation and risk of thromboembolism [69].

## 11. Possible Approaches to Acidosis Compensation

**Figure 4:**
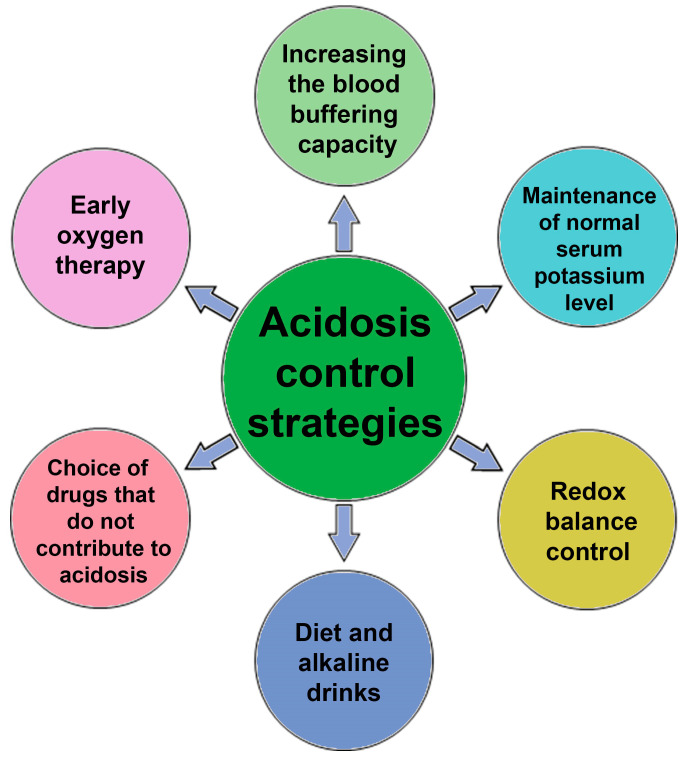
Scheme of possible interventions to combat acidosis in patients with COVID-19.

Considering the variety of cells and organs affected, the therapy of the disease and the prevention of the severe course of COVID-19 can be aimed at increasing the adaptive capabilities of the body. In the case of buffer systems, to compensate for acidosis (regardless of its metabolic or respiratory origin), appropriate drop solutions can be used to replenish the buffer capacity of blood and to normalize blood potassium levels [70]. At the same time, the excess content of glucose and lactate can be compensated using nicotinic acid derivatives that can be metabolized into NADP, which is a coenzyme in glycolysis. An increase in the concentration of NADPH leads to a decrease in the concentration of lactate, a shift in equilibrium toward accumulation of pyruvate and a reduced risk of developing lactic acidosis. Note that NADPH accelerates the reduction of oxidized glutathione, which leads to a decrease in the aggregation of erythrocytes and platelets, which can be due to oxidized sulfhydryl groups of membrane proteins [71] (Figure 4).

Acidosis can not only be diagnosed in intensive care units (acid-base balance analysis), but it can also be detected in publicly available clinical diagnostic laboratories (CDL). For example, simple measurements of lactate, glucose, total protein and albumin, K^+^, Na^+^, Cl^−^ ions, creatinine, and urea levels are informative.

The positive role of diet changes (restriction of carbohydrates, fats, spices) and adherence to diet therapy in the prevention of the severe course of COVID-19 should also be noted. Another obvious and most important way to prevent acidosis is early oxygen intervention and maintenance of blood oxygen saturation in patients. It helps to avoid the metabolic switch to anaerobic glycolysis.

A particular way of preventing acidosis is the intake of alkaline drinks, which is used by athletes, runners, and cyclists. Usually, sodium and potassium citrate are taken. The same method is used to dissolve some types of kidney stones. However, one must be aware that direct correction of blood pH with alkaline solutions is dangerous, since it affects a variety of processes and, in general, can increase mortality among critical patients.

Determination of urine pH and blood oxygenation may indirectly indicate the state of the bicarbonate buffer; they can be recommended for self-diagnosis during the initial period of the disease. Metabolic acidosis is associated with several aggravating factors, including fat oxidation inhibition and macrophage reprogramming [72,73,74].

Attention should be paid to drugs that provoke the development of metabolic acidosis, for example, metformin [75] and ibuprofen [76].

## 12. Hypotheses of COVID-19 Related Acidosis

Several studies have presented hypotheses linking COVID-19 and acidosis. Liao et al. [77] considered the effect of renin-angiotensin-aldosterone system (RAAS) inhibitors on the course of COVID-19. The authors suggested that the virus, through interaction with ACE2 receptors, can affect the RAAS and thereby disrupt the normal regulation of pH by the kidneys (proton excretion). As a result of damage of tissue by the SARS-CoV-2 virus, the total number of these receptors may decrease due to the massive death of cells containing many ACE2 receptors. If this hypothesis is correct, the virus can directly cause acidosis, and this regulation should be added to the scheme on Figure 1. Letarov and co-authors proposed that the particles released because of spontaneous activation of S1 spikes can bind to the receptor and cause a decrease in the representation of ACE2 on uninfected cells, which can lead to a local imbalance of the renin-angiotensin system and even greater activation of thrombosis [78].

It is important to note that the conformation of the S-protein of SARS-CoV-2 can be sensitive to changes in pH [79]. There are direct experimental data indicating a decrease in the affinity of protective antibodies to the S-protein at lower pH. Although in the blood plasma in the lungs the pH varies in the range of 7.2–7.6, locally in the parts of the lung affected by the virus, there may be a blood clot, a violation of blood circulation, and decreased gas exchange. Arising local acidosis can lead to a lactate concentration of 40 mM in the damaged tissue and thus result in significant pH shift in the focus of inflammation. Previously, it was hypothesized that a change in the S-protein conformation may lead to the virus escaping the immune response [80,81].

## 13. Discussion

Decreased oxygen levels in the blood are a key problem in COVID-19 patients. The main reason for a decrease in the level of oxygen saturation are damage to the lung tissue, leading to impaired ventilation, and the formation of microthrombi in the vessels of the lungs, causing a violation of the microcirculation of these vessels. The virus infection of immature red blood cells leads to a corresponding decrease in blood hemoglobin levels that contributes to hypoxia. At the molecular level, the lack of oxygen in the tissues prevents the oxidation of NADH, which leads to a decrease in the pH of the medium.

Due to the Bohr effect, it is possible to shift the equilibrium in the peripheral tissues toward the dissociation of oxyhemoglobin. The increasing deficiency of NAD^+^ in the glycolysis reaction does not allow the synthesis of 1,3-diphosphoglycerate with the subsequent formation of an ATP molecule. An increase in oxygen deficiency leads to the launch of the synthesis of 2,3-DPG, a molecule that stabilizes hemoglobin in deoxy form, which also improves the supply of oxygen to tissues. Ongoing oxygen deficiency triggers the reduction of pyruvate to lactate in order to regenerate NADH to NAD^+^. Excess lactate, a stronger acid than pyruvate, leads to increased acidosis. This is observed when the possibility of depositing this acid by cells is exhausted. Further, with the progression of oxygen deficiency, the pH value of the blood decreases, which in turn impairs the delivery of oxygen to peripheral tissues. The “point” of hemoglobin unloading with oxygen is not the “end point of the path”—peripheral tissues, but an intermediate section of oxygen transport from the lungs to the tissues.

**Figure 5:**
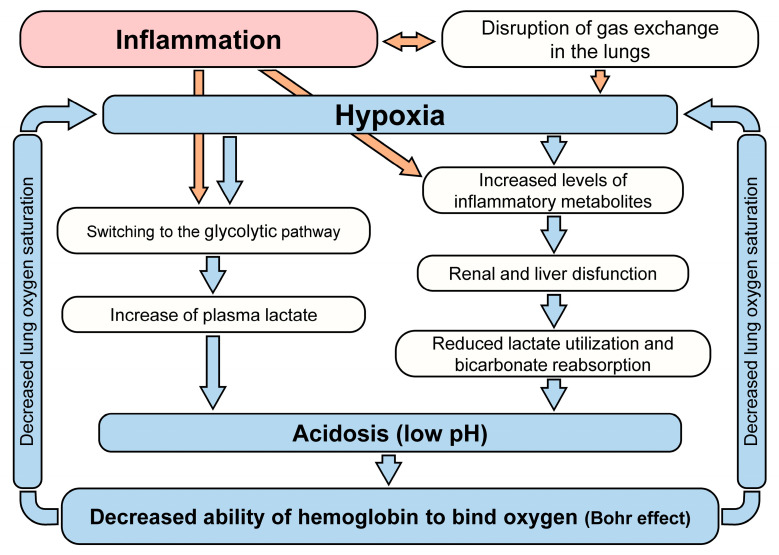
A diagram illustrating the system of direct and inverse relationships between inflammation, hypoxia, and saturation.

Hypoxia and acidosis reinforce each other. Figure 5 provides a general outline highlighting the vicious circle linking hypoxia and acidosis.

As COVID-19 progresses, causes of both metabolic and respiratory acidosis emerge. Depletion of the buffering capacity of the blood, depletion of the body’s resources necessary to contain the developing acidosis, is critical for reducing saturation. However, the risk groups (see above) are united by the degree of proximity to metabolic acidosis, since respiratory acidosis does not manifest itself before the development of respiratory disease. For example, we can discuss respiratory acidosis in patients with chronic obstructive pulmonary disease (COPD), but these patients also have all the signs of metabolic acidosis [82].

In the text of our review, attention is paid to metabolic acidosis and the possible role of lactate in the pathogenesis of COVID-19. However, we do not perceive lactate as the only cause of acidosis.

Acidosis develops when the buffer capacity of the blood is depleted and a striking example of this is the development of acidosis in diarrhea, briefly discussed above.

Another source of acidosis can be hypercapnia, an excess of carbon dioxide in the blood. Disruption of gas exchange in the lungs of patients with COVID-19 creates favorable conditions for the occurrence of hypercapnia and the development of respiratory acidosis. It is possible that the development of respiratory acidosis up to a certain point inhibits the growth of lactate. Experiments are described in which hypercapnic therapy (elevation of carbon dioxide levels in the blood) leads to a decrease in lactate [83,84,85].

Respiratory and metabolic acidosis are manifested in different ways in the dynamics of the biochemical parameters of the blood, but in both cases the body is forced to spend resources on compensating for acidosis and, on protecting the lungs from the possibility of developing acidosis as well as from a drop in saturation.

## 14. Conclusions

We have examined several ways in which hypoxia and acidosis affect the development of severe COVID-19. This effect has been described at the levels of the organism, organs, tissues, cells, and proteins: from the compensatory regulation of acidosis by the human body to the functioning of a single hemoglobin molecule. Several mechanisms linking the damaging factors of COVID-19 with acidosis are described. These mechanisms have a trigger, step-by-step character of action with pronounced positive feedback. A drop in blood oxygen saturation due to a decrease in blood pH in accordance with Bohr effect is a characteristic feature of the severe course of the disease caused by SARS-CoV-2. This drop is a consequence of the depletion of the compensatory capabilities of the body to regulate acidosis. COVID-19 disease has a systemic damaging effect on various organs and tissues. Among them are DIC-syndrome, pneumonia, and nervous system damage. This disease leads to a variety of different complications; it seems to check the body for the presence of “weak points” and regulatory circuits that have little stability. It is quite possible that acidosis must be compensated for during the rehabilitation of these patients.

Although all the listed triggers that accelerate the onset of acidosis can be significant in other diseases, the combination of all these effects in one disease looks like a unique phenomenon. It should be emphasized that hypoxia and acidosis make it possible to bring the contribution of all these mechanisms to one “common denominator”. Ultimately, acidosis is a consequence of a decrease in blood oxygen saturation; however, it contributes to this decrease.

A feature of the pathogenesis of severe COVID-19 is the simultaneous presence of an infectious process and trauma (vascular damage) [86]. Treatment of these two conditions involves alternative activities of the immune system, the main regulators of which are macrophages. It is possible that the problem lies precisely in the fact that the immune system is trying “to kill two birds with one stone”, constantly and inefficiently switching between different types of responses. The disease persists; destruction and inflammation are growing.

Along with other colleagues [70], we believe that to prevent severe cases of COVID-19, increased attention should be paid to the diagnosis and possible relief of acidosis.

## Data Availability

All the results found are available in this manuscript.

## References

[1] Chhetri S., Khamis F., Pandak N., Al Khalili H., Said E., Petersen E. (2020). A fatal case of COVID-19 due to metabolic acidosis following dysregulate inflammatory response (cytokine storm). IDCases.

[2] Lodyagin A., Batotsyrenov B., Shikalova I., Voznyuk I. (2020). Acidosis and toxic hemolysis-goals of pathogenetic treatment of polyorgan pathology in COVID-19. Bull. Rehabil. Med..

[3] Shevel E. (2020). Conditions favoring increased COVID-19 morbidity and mortality: Their common denominator and treatment. Isr. Med. Assoc. J. IMAJ.

[4] Price-Haywood E.G., Burton J., Fort D., Seoane L. (2020). Hospitalization and mortality among black patients and white patients with Covid-19. N. Engl. J. Med..

[5] Vassiliou A.G., Jahaj E., Ilias I., Markaki V., Malachias S., Vrettou C., Ischaki E., Mastora Z., Douka E., Keskinidou C. (2020). Lactate kinetics reflect organ dysfunction and are associated with adverse outcomes in intensive care unit patients with COVID-19 pneumonia: Preliminary results from a GREEK Single-Centre Study. Metabolites.

[6] Velavan T.P., Kieu Linh L.T., Kreidenweiss A., Gabor J., Krishna S., Kremsner P.G. (2021). Longitudinal monitoring of lactate in hospitalized and ambulatory COVID-19 patients. Am. J. Trop. Med. Hyg..

[7] Newington J.T., Harris R.A., Cumming R.C. (2013). Reevaluating metabolism in Alzheimer’s disease from the perspective of the astrocyte-neuron lactate shuttle model. J. Neurodegener. Dis..

[8] Andersen L.W., Mackenhauer J., Roberts J.C., Berg K.M., Cocchi M.N., Donnino M.W. (2013). Etiology and therapeutic approach to elevated lactate levels. Mayo Clin. Proc..

[9] Kraut J.A., Madias N.E. (2014). Lactic acidosis. N. Engl. J. Med..

[10] Ma L.N., Huang X.B., Muyayalo K.P., Mor G., Liao A.H. (2020). Lactic acid: A novel signaling molecule in early pregnancy?. Front. Immunol..

[11] Li J., Wang X., Chen J., Zuo X., Zhang H., Deng A. (2020). COVID-19 infection may cause ketosis and ketoacidosis. Diabetes Obes. Metab..

[12] Kraut J.A., Madias N.E. (2010). Metabolic acidosis: Pathophysiology, diagnosis and management. Nat. Rev. Nephrol..

[13] Chycki J., Kurylas A., Maszczyk A., Golas A., Zajac A. (2018). Alkaline water improves exercise-induced metabolic acidosis and enhances anaerobic exercise performance in combat sport athletes. PLoS ONE.

[14] Pillai S., Davies G., Lawrence M., Whitley J., Stephens J., Williams P.R., Morris K., Evans P.A. (2021). The effect of diabetic ketoacidosis (DKA) and its treatment on clot microstructure: Are they thrombogenic?. Clin. Hemorheol. Microcirc..

[15] Chee Y.J., Tan S.K., Yeoh E. (2020). Dissecting the interaction between COVID-19 and diabetes mellitus. J. Diabetes Investig..

[16] Orioli L., Hermans M.P., Thissen J.P., Maiter D., Vandeleene B., Yombi J.C. (2020). COVID-19 in diabetic patients: Related risks and specifics of management. Ann. Endocrinol..

[17] Palermo N.E., Sadhu A.R., McDonnell M.E. (2020). Diabetic ketoacidosis in COVID-19: Unique concerns and considerations. J. Clin. Endocrinol. Metab..

[18] Gentile S., Strollo F., Mambro A., Ceriello A. (2020). COVID-19, ketoacidosis and new-onset diabetes: Are there possible cause and effect relationships among them?. Diabetes Obes. Metab..

[19] Rubino F., Amiel S.A., Zimmet P., Alberti G., Bornstein S., Eckel R.H., Mingrone G., Boehm B., Cooper M.E., Chai Z. (2020). New-onset diabetes in Covid-19. N. Engl. J. Med..

[20] Cecere N., Hubinont C., Kabulu Kadingi A., Vincent M.F., Van den Bergh P., Onnela A., Hantson P. (2013). Extreme maternal metabolic acidosis leading to fetal distress and emergency caesarean section. Case Rep. Obstet. Gynecol..

[21] Mason R.J. (2020). Pathogenesis of COVID-19 from a cell biology perspective. Eur. Respir. J..

[22] Ackermann M., Verleden S.E., Kuehnel M., Haverich A., Welte T., Laenger F., Vanstapel A., Werlein C., Stark H., Tzankov A. (2020). Pulmonary vascular endothelialitis, thrombosis, and angiogenesis in COVID-19. N. Engl. J. Med..

[23] Hellman U., Karlsson M.G., Engstrom-Laurent A., Cajander S., Dorofte L., Ahlm C., Laurent C., Blomberg A. (2020). Presence of hyaluronan in lung alveoli in severe Covid-19: An opening for new treatment options?. J. Biol. Chem..

[24] Shahbaz S., Xu L., Osman M., Sligl W., Shields J., Joyce M., Tyrrell D.L., Oyegbami O., Elahi S. (2021). Erythroid precursors and progenitors suppress adaptive immunity and get invaded by SARS-CoV-2. Stem Cell Rep..

[25] Lunt S.Y., Vander Heiden M.G. (2011). Aerobic glycolysis: Meeting the metabolic requirements of cell proliferation. Annu. Rev. Cell Dev. Biol..

[26] Borregaard N., Schwartz J.H., Tauber A.I. (1984). Proton secretion by stimulated neutrophils. Significance of hexose monophosphate shunt activity as source of electrons and protons for the respiratory burst. J. Clin. Investig..

[27] Niederman R., Zhang J., Kashket S. (1997). Short-chain carboxylic-acid-stimulated, PMN-mediated gingival inflammation. Crit. Rev. Oral Biol. Med..

[28] Erra Diaz F., Dantas E., Geffner J. (2018). Unravelling the interplay between extracellular acidosis and immune cells. Mediat. Inflamm..

[29] Semenza G.L. (2000). HIF-1: Mediator of physiological and pathophysiological responses to hypoxia. J. Appl. Physiol..

[30] Ivashkiv L.B. (2020). The hypoxia-lactate axis tempers inflammation. Nat. Rev. Immunol..

[31] Serebrovska Z.O., Chong E.Y., Serebrovska T.V., Tumanovska L.V., Xi L. (2020). Hypoxia, HIF-1alpha, and COVID-19: From pathogenic factors to potential therapeutic targets. Acta Pharmacol. Sin..

[32] Zhang D., Tang Z., Huang H., Zhou G., Cui C., Weng Y., Liu W., Kim S., Lee S., Perez-Neut M. (2019). Metabolic regulation of gene expression by histone lactylation. Nature.

[33] Khatib-Massalha E., Bhattacharya S., Massalha H., Biram A., Golan K., Kollet O., Kumari A., Avemaria F., Petrovich-Kopitman E., Gur-Cohen S. (2020). Lactate released by inflammatory bone marrow neutrophils induces their mobilization via endothelial GPR81 signaling. Nat. Commun..

[34] Popel A.S. (1989). Theory of oxygen transport to tissue. Crit. Rev. Biomed. Eng..

[35] Werigo B. (1892). Zur Frage über die Wirkung des Sauerstoffs auf die Kohlensäureausscheidung in den Lungen. Arch. Gesamte Physiol. Menschen Tiere.

[36] Bohr C., Hasselbalch K., Krogh A. (1904). Concerning a biologically important relationship–the influence of the carbon dioxide content of blood on its oxygen binding. Skand. Arch. Physiol..

[37] Gell D.A. (2018). Structure and function of haemoglobins. Blood Cells Mol. Dis..

[38] Ahmed M.H., Ghatge M.S., Safo M.K. (2020). Hemoglobin: Structure, function and allostery. Subcell. Biochem..

[39] Srinivasan A.J., Morkane C., Martin D.S., Welsby I.J. (2017). Should modulation of p50 be a therapeutic target in the critically ill?. Expert Rev. Hematol..

[40] Stewart T., Lambourne J., Thorp-Jones D., Thomas D.W. (2021). Implementation of early management of iron deficiency in pregnancy during the SARS-CoV-2 pandemic. Eur. J. Obstet. Gynecol. Reprod. Biol..

[41] Kislyakov Y.Y., Breslav I.S. (1988). Respiration, Gas Dynamics and Performance in Hyperbaria.

[42] Storz J.F., Moriyama H. (2008). Mechanisms of hemoglobin adaptation to high altitude hypoxia. High Alt. Med. Biol..

[43] Kislyakov Y.Y. (1987). O_2_ transport mechanisms in the microcirculation system. Physiol. J. USSR.

[44] Tusman G., Bohm S.H., Suarez-Sipmann F., Scandurra A., Hedenstierna G. (2010). Lung recruitment and positive end-expiratory pressure have different effects on CO_2_ elimination in healthy and sick lungs. Anesth. Analg..

[45] Zaitseva A.Y., Kislyakov Y.Y., Masing M.S., Davydov V.V. (2020). Application of a non-invasive optical learning diagnostic system and mathematical methods for analyzing multidimensional data to assess the oxygen status of human tissues. Sci. Instrum..

[46] Dhont S., Derom E., Van Braeckel E., Depuydt P., Lambrecht B.N. (2020). The pathophysiology of ‘happy’ hypoxemia in COVID-19. Respir. Res..

[47] Asakura H., Ogawa H. (2021). COVID-19-associated coagulopathy and disseminated intravascular coagulation. Int. J. Hematol..

[48] Mosleh W., Chen K., Pfau S.E., Vashist A. (2020). Endotheliitis and endothelial dysfunction in patients with COVID-19: Its role in thrombosis and adverse outcomes. J. Clin. Med..

[49] Hottz E.D., Azevedo-Quintanilha I.G., Palhinha L., Teixeira L., Barreto E.A., Pao C.R.R., Righy C., Franco S., Souza T.M.L., Kurtz P. (2020). Platelet activation and platelet-monocyte aggregate formation trigger tissue factor expression in patients with severe COVID-19. Blood.

[50] Iba T., Levy J.H., Levi M., Thachil J. (2020). Coagulopathy in COVID-19. J. Thromb. Haemost..

[51] Engstrom M., Schott U., Romner B., Reinstrup P. (2006). Acidosis impairs the coagulation: A thromboelastographic study. J. Trauma Inj. Infect. Crit. Care.

[52] Engstrom M., Schott U., Nordstrom C.H., Romner B., Reinstrup P. (2006). Increased lactate levels impair the coagulation system--a potential contributing factor to progressive hemorrhage after traumatic brain injury. J. Neurosurg. Anesthesiol..

[53] Tang N., Li D., Wang X., Sun Z. (2020). Abnormal coagulation parameters are associated with poor prognosis in patients with novel coronavirus pneumonia. J. Thromb. Haemost..

[54] Kobayashi S., Gando S., Morimoto Y., Nanzaki S., Kemmotsu O. (2001). Serial measurement of arterial lactate concentrations as a prognostic indicator in relation to the incidence of disseminated intravascular coagulation in patients with systemic inflammatory response syndrome. Surg. Today.

[55] Vanni S., Jimenez D., Nazerian P., Morello F., Parisi M., Daghini E., Pratesi M., Lopez R., Bedate P., Lobo J.L. (2015). Short-term clinical outcome of normotensive patients with acute PE and high plasma lactate. Thorax.

[56] Zabczyk M., Natorska J., Janion-Sadowska A., Malinowski K.P., Janion M., Undas A. (2020). Elevated lactate levels in acute pulmonary embolism are associated with prothrombotic fibrin clot properties: Contribution of NETs formation. J. Clin. Med..

[57] D’Amico F., Baumgart D.C., Danese S., Peyrin-Biroulet L. (2020). Diarrhea during COVID-19 infection: Pathogenesis, epidemiology, prevention, and management. Clin. Gastroenterol. Hepatol..

[58] Xu J., Chu M., Zhong F., Tan X., Tang G., Mai J., Lai N., Guan C., Liang Y., Liao G. (2020). Digestive symptoms of COVID-19 and expression of ACE2 in digestive tract organs. Cell Death Discov..

[59] Gennari F.J., Weise W.J. (2008). Acid-base disturbances in gastrointestinal disease. Clin. J. Am. Soc. Nephrol..

[60] Booth A.L., Abels E., McCaffrey P. (2021). Development of a prognostic model for mortality in COVID-19 infection using machine learning. Mod. Pathol..

[61] Poole D.C., Rossiter H.B., Brooks G.A., Gladden L.B. (2021). The anaerobic threshold: 50+ years of controversy. J. Physiol..

[62] Hogan M.C. (2021). What Wasserman wrought: A celebratory review of 50 years of research arising from the concept of an ‘anaerobic threshold’. J. Physiol..

[63] Rabinowitz J.D., Enerback S. (2020). Lactate: The ugly duckling of energy metabolism. Nat. Metab..

[64] Dietl K., Renner K., Dettmer K., Timischl B., Eberhart K., Dorn C., Hellerbrand C., Kastenberger M., Kunz-Schughart L.A., Oefner P.J. (2010). Lactic acid and acidification inhibit TNF secretion and glycolysis of human monocytes. J. Immunol..

[65] Suzuki H., Hisamatsu T., Chiba S., Mori K., Kitazume M.T., Shimamura K., Nakamoto N., Matsuoka K., Ebinuma H., Naganuma M. (2016). Glycolytic pathway affects differentiation of human monocytes to regulatory macrophages. Immunol. Lett..

[66] Cassim S., Pouyssegur J. (2019). Tumor microenvironment: A metabolic player that shapes the immune response. Int. J. Mol. Sci..

[67] Sun L., Yang X., Yuan Z., Wang H. (2020). Metabolic reprogramming in immune response and tissue inflammation. Arterioscler. Thromb. Vasc. Biol..

[68] Nolt B., Tu F., Wang X., Ha T., Winter R., Williams D.L., Li C. (2018). Lactate and immunosuppression in sepsis. Shock.

[69] Awasthi D., Nagarkoti S., Sadaf S., Chandra T., Kumar S., Dikshit M. (2019). Glycolysis dependent lactate formation in neutrophils: A metabolic link between NOX-dependent and independent NETosis. Biochim. Biophys. Acta BBA—Mol. Basis Dis..

[70] Quade B.N., Parker M.D., Occhipinti R. (2021). The therapeutic importance of acid-base balance. Biochem. Pharmacol..

[71] Coller B.S. (2005). Leukocytosis and ischemic vascular disease morbidity and mortality: Is it time to intervene?. Arterioscler. Thromb. Vasc. Biol..

[72] Castleberry A.W., Grannis F.W. (2010). What is a reasonable cost to refute a preposterous hypothesis?. Br. J. Cancer.

[73] Corbet C., Pinto A., Martherus R., Santiago de Jesus J.P., Polet F., Feron O. (2016). Acidosis drives the reprogramming of fatty acid metabolism in cancer cells through changes in mitochondrial and histone acetylation. Cell Metab..

[74] Ventura F.V., Ruiter J.P., IJlst L., de Almeida I.T., Wanders R.J. (1998). Lactic acidosis in long-chain fatty acid beta-oxidation disorders. J. Inherit. Metab. Dis..

[75] Cheng X., Liu Y.M., Li H., Zhang X., Lei F., Qin J.J., Chen Z., Deng K.Q., Lin L., Chen M.M. (2020). Metformin is associated with higher incidence of acidosis, but not mortality, in individuals with COVID-19 and pre-existing type 2 diabetes. Cell Metab..

[76] Downie A., Ali A., Bell D. (1993). Severe metabolic acidosis complicating massive ibuprofen overdose. Postgrad. Med. J..

[77] Liao W.H., Yang G.G., Henneberg M. (2020). The renin-angiotensin-aldosterone system inhibitors in COVID-19: From acidosis to ventilation and immunity. Swiss Med. Wkly..

[78] Letarov A.V., Babenko V.V., Kulikov E.E. (2021). Free SARS-CoV-2 spike protein S1 particles may play a role in the pathogenesis of COVID-19 infection. Biochemistry.

[79] Zhou T., Tsybovsky Y., Gorman J., Rapp M., Cerutti G., Chuang G.Y., Katsamba P.S., Sampson J.M., Schon A., Bimela J. (2020). Cryo-EM structures of SARS-CoV-2 spike without and with ACE2 reveal a pH-dependent switch to mediate endosomal positioning of receptor-binding domains. Cell Host Microbe.

[80] Nechipurenko Y.D., Anashkina A.A., Matveeva O.V. (2020). Change of antigenic determinants of SARS-CoV-2 virus S-protein as a possible cause of antibody-dependent enhancement of virus infection and cytokine storm. Biophysics.

[81] Zaichuk T.A., Nechipurenko Y.D., Adzhubey A.A., Onikienko S.B., Chereshnev V.A., Zainutdinov S.S., Kochneva G.V., Netesov S.V., Matveeva O.V. (2020). The challenges of vaccine development against Betacoronaviruses: Antibody dependent enhancement and Sendai virus as a possible vaccine vector. Mol. Biol..

[82] Bruno C.M., Valenti M. (2012). Acid-base disorders in patients with chronic obstructive pulmonary disease: A pathophysiological review. J. Biomed. Biotechnol..

[83] Engel K., Kildeberg P.A., Fine B.P., Winters R.W. (1967). Effects of acute respiratory acidosis on blood lactate concentration. Scand. J. Clin. Lab. Investig..

[84] McLellan T.M. (1991). The influence of a respiratory acidosis on the exercise blood lactate response. Eur. J. Appl. Physiol. Occup. Physiol..

[85] Kato T., Tsukanaka A., Harada T., Kosaka M., Matsui N. (2005). Effect of hypercapnia on changes in blood pH, plasma lactate and ammonia due to exercise. Eur. J. Appl. Physiol..

[86] Lupu L., Palmer A., Huber-Lang M. (2020). Inflammation, thrombosis, and destruction: The three-headed cerberus of trauma- and SARS-CoV-2-induced ARDS. Front. Immunol..

